# Advances in Retinal Optical Imaging

**DOI:** 10.3390/photonics5020009

**Published:** 2018-04-27

**Authors:** Yanxiu Li, Xiaobo Xia, Yannis M. Paulus

**Affiliations:** 1 Department of Ophthalmology and Visual Sciences, University of Michigan, Ann Arbor, MI 48105, USA;; 2 Department of Ophthalmology, Xiangya Hospital, Central South University, Changsha 410008, China;; 3 Department of Biomedical Engineering, University of Michigan, Ann Arbor, MI 48015, USA

**Keywords:** optical imaging, retina, optical coherence tomography (OCT), optical coherence tomography angiography (OCTA), photoacoustic microscopy, fundus autofluorescence, adaptive optics (AO), scanning laser ophthalmoscopy (SLO), molecular imaging, age-related macular degeneration (AMD), diabetic retinopathy (DR), retinal vein occlusions (RVO), choroidal neovascularization (CNV)

## Abstract

Retinal imaging has undergone a revolution in the past 50 years to allow for better understanding of the eye in health and disease. Significant improvements have occurred both in hardware such as lasers and optics in addition to software image analysis. Optical imaging modalities include optical coherence tomography (OCT), OCT angiography (OCTA), photoacoustic microscopy (PAM), scanning laser ophthalmoscopy (SLO), adaptive optics (AO), fundus autofluorescence (FAF), and molecular imaging (MI). These imaging modalities have enabled improved visualization of retinal pathophysiology and have had a substantial impact on basic and translational medical research. These improvements in technology have translated into early disease detection, more accurate diagnosis, and improved management of numerous chorioretinal diseases. This article summarizes recent advances and applications of retinal optical imaging techniques, discusses current clinical challenges, and predicts future directions in retinal optical imaging.

## 1. Introduction

The human eye is optically transparent, and thus the retina and choroid are ideally suited for optical imaging of pathological disease changes. By the end of the 19th century, the first photograph of the human retina had been obtained [1]. Since then, there have been huge advances in ophthalmic imaging, including fluorescein angiography (FA) and indocyanine green angiography (ICGA) and more recently scanning laser ophthalmoscopy (SLO) and optical coherence tomography (OCT). The development of these optical instruments has greatly extended our ability to evaluate chorioretinal disease pathophysiology. Furthermore, OCT angiography (OCTA) as a noninvasive angiographic technique provides visualization of vascular structures. Other appealing technologies such as adaptive optics (AO), ultra-wide field imaging, fundus autofluorescence (FAF), and photoacoustic microscopy (PAM) have been integrated into available retinal imaging modalities, which can significantly improve our image quality or field of view. Molecular imaging (MI) is an area that combines retinal molecular biomarkers and advanced ocular imaging technologies and has the potential to visualize the earliest cellular and biochemical process before advanced retinal disease. This review focuses on emerging imaging instruments, their recent advances, and medical applications.

## 2. Optical Coherence Tomography (OCT)

Optical coherence tomography (OCT) was first introduced for ophthalmic imaging in 1991 [2], and OCT has been rapidly and widely adopted in ophthalmology [3]. As the eye is optically accessible for visible and near-infrared light, ophthalmic OCT allows an unparalleled combination of high axial resolution (1–10 μm) with appropriate tissue penetration depth (1–2 mm in tissue) [4]. Fourier-domain OCT (FD-OCT) can be characterized into two types: spectral-domain OCT (SD-OCT) and swept source OCT (SS-OCT). With the development of time-domain OCT (TD-OCT) and the more recent FD-OCT, high quality images of the retinal structure are now clearly demonstrated, such as inner limiting membrane (ILM), retinal nerve fiber layer (RNFL), photoreceptor inner and outer segments (IS/OS), and outer limiting membrane [5]. Even though SD-OCT and SS-OCT both use the Fourier transform, SD-OCT instruments use a broadband near infrared superluminescent diode as a light source with a spectrometer as the detector. Current clinical systems often employ a center wavelength of about 840 nm (e.g., Carl Zeiss Meditec, Inc., Jena, Germany). SS-OCT instruments use a tunable swept laser, with a single photodiode detector. Current clinical systems often employ a longer center wavelength of about 1050 nm [6,7]. TD-OCT technology has limited speed and sensitivity compared with more recent SD-OCT and SS-OCT. SD-OCT has greatly improved speed and sensitivity and is able to detect small changes in morphology of retinal layers and choroidal neovascularization (CNV) activity. However, SD-OCT has difficulty differentiating vascular and fibrous components of CNV because of their similar reflectivity properties [8,9].

SS-OCT technology is the latest development in OCT technology. SS-OCT often utilizes infrared (1050 nm) laser source, has less susceptibility to sensitivity roll-off, and has ultrahigh speed image acquisition often of 100,000 Hz A scans speed or higher for clinical systems and above 1 MHz in research systems [10–14]. These characteristics of SS-OCT enable deeper penetration, excellent axial resolution, and fewer motion artifacts, to generate ultrahigh-definition B-scan images of the retinal microstructure [9]. For example, in patients with classic choroidal neovascularization (CNV), SS-OCT images in line scan mode can show intraretinal edema, CNV lesions, outer retina disorganization, RPE atrophy, and choroid and choriocapillaris thickness. SS-OCT *en face* mode provides a coronal view of the posterior segment of different depths and enables visualization of structures that cannot be easily appreciated in cross-sectional images [15]. When B-scan and *en face* mode are used together, they may provide additional anatomic insight into diseases in a non-invasive manner. The Topcon DRI Triton and the Zeiss Plex Elite 9000 are two clinical systems based on SS-OCT technology. They also provide 3D volumetric cubes that can be viewed either in cross-sectional or *en face* views [16–18]. Ferrara et al. [15] have described *en face* SS-OCT findings in patients with chronic central serous chorioretinopathy (CSCR) and neovascular age-related macular degeneration (AMD) and have reported two distinct patterns of choroidal vascular dilatation: focal and diffuse. Dansingani et al. [17] used *en face* SS-OCT to correlate clinical manifestations with choroidal morphology in pachychoroid disorders, including central serous chorioretinopathy, pachychoroid pigment epitheliopathy, pachychoroid neovasculopathy, and polypoidal choroidal vasculopathy. They find that *en face* SS-OCT can localize the changes of increased thickness and dilated outer choroidal vessels at disease foci. Dastiridou et al. [19] uses a SS-OCT device (DRI-OCT1 Atlantis; Topcon) to characterize choroidal thickness and choroidal reflectivity in the eyes of patients with birdshot chorioretinopathy. They found that there are evident changes in choroidal reflectivity and choroidal thickness between active and inactive birdshot chorioretinopathy patients. A study by Lee et al. [20] investigated total retinal blood flow(TRBF) in diabetic eyes with or without diabetic retinopathy (DR) and in healthy eyes using the high-speed *en face* Doppler OCT. They demonstrate that diabetic eyes with DME exhibited lower TRBF than healthy eyes. *En face* Doppler OCT provides an effective method for measuring TRBF in the clinic.

## 3. OCT Angiography (OCTA)

OCT angiography (OCTA) is a new, noninvasive imaging technique based on OCT imaging which allows for the visualization of the retinal and choroidal microvasculature without the injection of exogenous dyes [3,21–25]. OCTA is a method of visualizing vasculature that is enhanced from the signal (intensity and/or phase) change caused by erythrocyte movement that arises from multiple B-scans performed at the same position. OCTA images are essentially motion-contrast images.

Various algorithms have been developed for OCTA devices. OCTA can be separated into three categories: phase-signal-based OCTA (e.g., Doppler OCT, phase-variance OCT), intensity-signal-based OCTA (e.g., speckle-variance OCT, correlation mapping OCT, OCTA ratio analysis and split-spectrum amplitude decorrelation angiography), and complex-signal-based OCTA (e.g., optical microangiography, multiple signal classification OMAG, and imaginary part-based correlation mapping OCT) [26]. Direct comparisons among OCTA algorithms are currently limited [27,28]. In 2012, Jia et al. [29] proposed the split-spectrum amplitude decorrelation angiography (SSADA) algorithm, as a kind of intensity-signal-based OCTA method, based on the decorrelation of OCT signal amplitude due to flow. It has been demonstrated that the SSADA algorithm improves the signal to noise ratio of flow direction and facilitates visualization of retinal vasculature [8,29]. OCTA ratio analysis (OCTARA) is the intensity ratio calculation OCTA algorithm. As the full spectrum is preserved, there is no loss of axial resolution [27,30]. Optical Microangiography (OMAG) algorithm [31–35] is a complex-signal-based OCTA technique, proposed first by Wang et al. [34] in 2007. It utilizes both the intensity and phase information of the OCT signal in the flow signal calculation to increase its sensitivity. OMAG can provide angiography either with or without directional information. Optical microangiography has been used to illustrate the microcirculation in human retina. In the United States, two commercially available OCTA systems currently approved by the Food and Drug Administration (FDA) use different algorithms. The AngioVue system (Optovue, Inc., Fremont, CA, USA) [36] uses the SSADA algorithm, while the Zeiss AngioPlex system (Carl Zeiss Meditec Inc., Dublin, CA, USA) [37] employs the OMAG algorithm.

**Figure 1.**
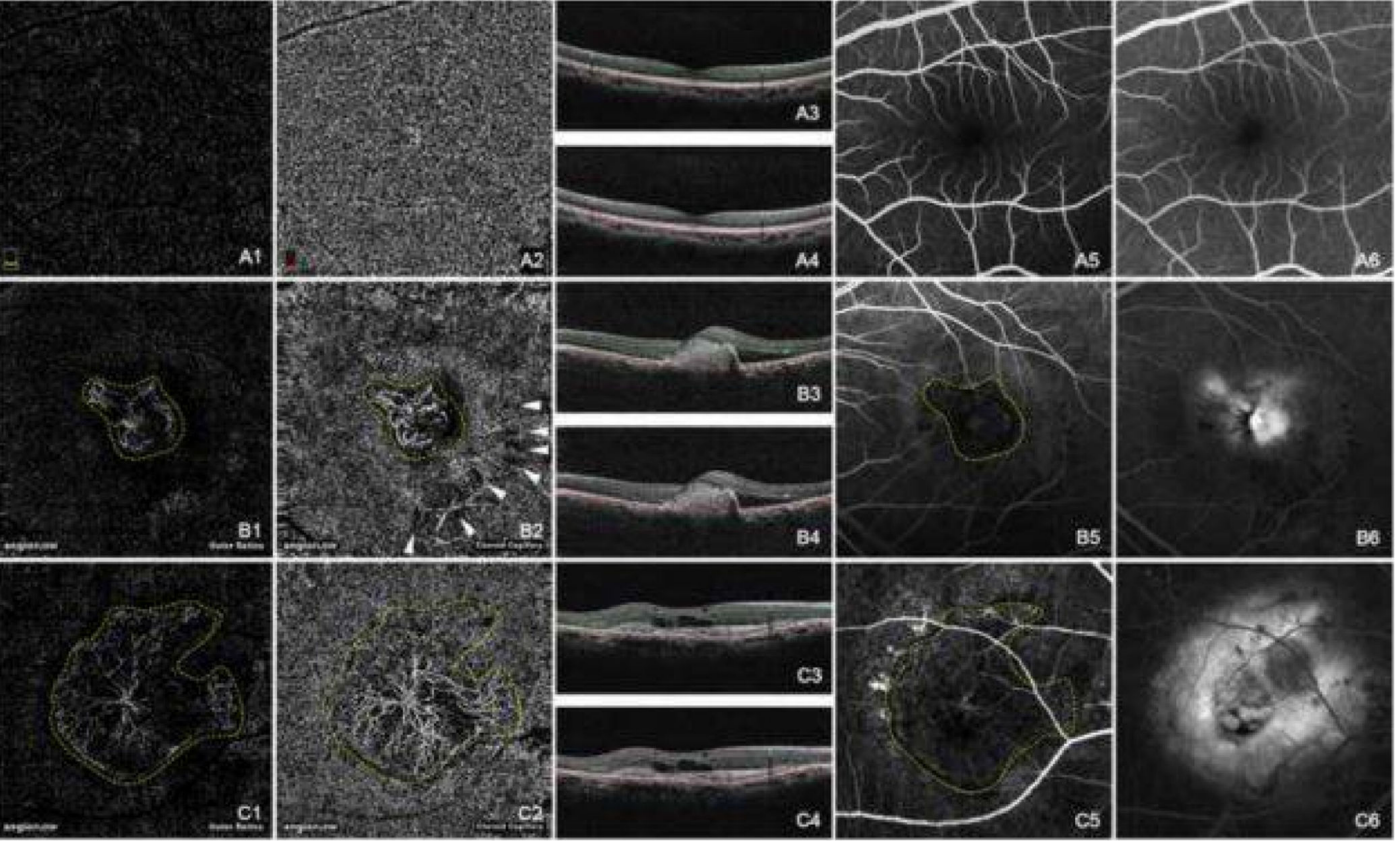
Normal choroidal vasculature (A) and CNV (B and C) demonstrated by OCTA and FA. The normal appearance of the outer retina shows an absence of blood flow (**A1**). The normal appearance of the choroid shows homogeneous grayish capillary beds (**A2**). The early phase and the late phase of normal FA images were shown in (**A5**) and (**A6**) respectively. Hyperreflective CNVs were demonstrated at both the outer retina level (**B1**,**C1**) and the choroid capillary level (**B2**,**C2**). The configurations of these CNV lesions were strikingly identical with those in the early phase of FA (**B5**,**C5**). CNV was in a globular aspect in (**B1**,**B2**), while in a fan-like shape with a feeder vessel in (**C1**,**C2**). The hyporeflective speckles (white arrowheads, **B2**) regarding the hard exudates were “mirror images” projected from the deep retinal plexus layer (not shown). The early phase of FA (**B5**,**C5**) revealed hyperfluorescent CNVs, while the late phase of FA (**B6**,**C6**) showed leakage and edema caused by CNV. The double lines in B scan OCT images of **A3**, **A4**, **B3**, **B4**, **C3**, and **C4** indicated the layers exhibited in **A1**, **A2**, **B1**, **B2**, **C1**, and **C2** respectively. (Yellow dashed line: CNV; White arrow head: “mirror image” of hard exudate) [5].

Clinical investigations that use OCTA have increased exponentially in recent years. OCTA has often replaced fluorescein angiography and become an important imaging method to evaluate retinal vascular diseases, including diabetic retinopathy (DR), retinal vein occlusions (RVO), and neovascular AMD (Figure 1) [38–41]. One common retinal vascular disease utilizing OCTA is to assess the microvascular pathology in DR [41–43]. OCTA can clearly visualize and quantify nonperfusion, neovascularization, and the foveal avascular zone (FAZ) area change, which can be helpful to identify non-proliferative DR and macular ischemia in patients with DR [44–46]. Hirano et al. [47] reported that SS-OCTA with extended field imaging (EFI) allows acquisition of large areas of *en face* images of retinal vasculature in patients with DR that are larger by an average ratio of 1.80 ± 0.18 (range, 1.50–2.18). OCTA is a noninvasive, rapid, and reliable method to evaluate the area of capillary nonperfusion and FAZ morphology in patients with RVO [48,49]. In addition, OCTA provides depth-resolved information that has never before been available with conventional fluorescein angiography. OCTA thus allows visualization of occult type 1 neovascular membranes in AMD, which are located under the RPE, and the microvascular details of which are not easily identified with conventional angiography [50]. Roisman et al. [51] demonstrated that SS-OMAG OCTA can identify type 1 neovascularization within ICGA plaques. One advantage of SS-OMAG OCTA over ICGA is that it can be easily repeated at follow-up visits because it is fast, safe, and noninvasive. OCTA can play an important role in treatment monitoring of intravitreal injections of anti-VEGF agents that are highly successful and currently the treatment of choice for neovascular AMD [52]. OCTA offers noninvasive monitoring of the retinal and choriocapillaris microvasculature in patients with CNV and can aid in treatment decisions during patient follow-up [53].

Fluorescein angiography (FA) is a vitally important diagnostic tool and has been the gold standard for the evaluation of patients with retinal disease since its advent in 1961 [54]. However, FA is an invasive test and requires typically 10–15 min to obtain images. FA also does not provide depth information and has difficulty imaging the deep capillary network well. OCTA, in comparison, is a non-invasive technique and has the capability to image all layers of the vasculature. However, it requires blood flow and lacks the ability to identify staining, leakage, and pooling [23,55]. OCTA technology also has other limitations. First, currently available clinical OCTA systems can have relatively poor axial resolution (~15 μm) due to signal averaging, limiting the identification of small-caliber vessels. Second, because OCTA uses the principle that movement in the back of the eye represents blood flow, it is prone to motion artifact. Although the presence of computer algorithms and eye tracking software are employed to help reduce motion artifacts, correction of all artifacts is challenging. In addition, OCTA creates flow images by comparing the differences between consecutive OCT B-scan images. OCTA may miss areas of slow blood flow such as in microaneurysms or fibrotic CNV, which give us very important information about diseases [27,55]. Future advances may reduce artifacts, and faster scanning speeds may help obtain larger fields of view. An efficient angiography algorithm with fast scanning speeds without sacrificing the posterior pole information would be a great boon to assessing the retinal vasculature non-invasively.

In conclusion, OCTA is a rapidly evolving, noninvasive, and dyeless technology that will potentially improve patient care by decreasing the disease morbidity through earlier disease detection and intervention.

## 4. Photoacoustic Microscopy (PAM)

Photoacoustic microscopy (PAM) is based on optical excitation and ultrasonic detection. A short pulse duration laser (nanosecond pulse duration) illuminates and excites a target tissue, thus inducing ultrasonic pressure waves because of specific optical absorption. The ultrasound transducer is focused on the tissue surface and records ultrasonic signals, generating an image [56–60].

Several groups have developed ocular PAM imaging systems [61–63]. As the technology has advanced, PAM can image retinal vasculature and the RPE with a better contrast-to-background ratio than any other intrinsic retinal imaging modality. One can obtain more quantitative imaging information, including estimation of oxygen saturation of hemoglobin (sO_2_) and retinal metabolic rate of oxygen (rMRO_2_) [64]. Hennen et al. [65] through PAM sO_2_ measurement concluded that PAM can resolve anatomic structures of the eye. PAM also provides a safe, non-invasive method of in vivo imaging of sO_2_, which is important to evaluate the role of oxidative damage, hypoxia, and ischemia in the pathogenesis of ocular diseases. Song et al. [66] combined PAM with SD-OCT to measure rMRO_2_ by having PAM measure the sO_2_ and SD-OCT map the blood flow rate. The quantitative method helps further understanding of some ocular diseases, including DR, RVO, and glaucoma.

**Figure 2.**
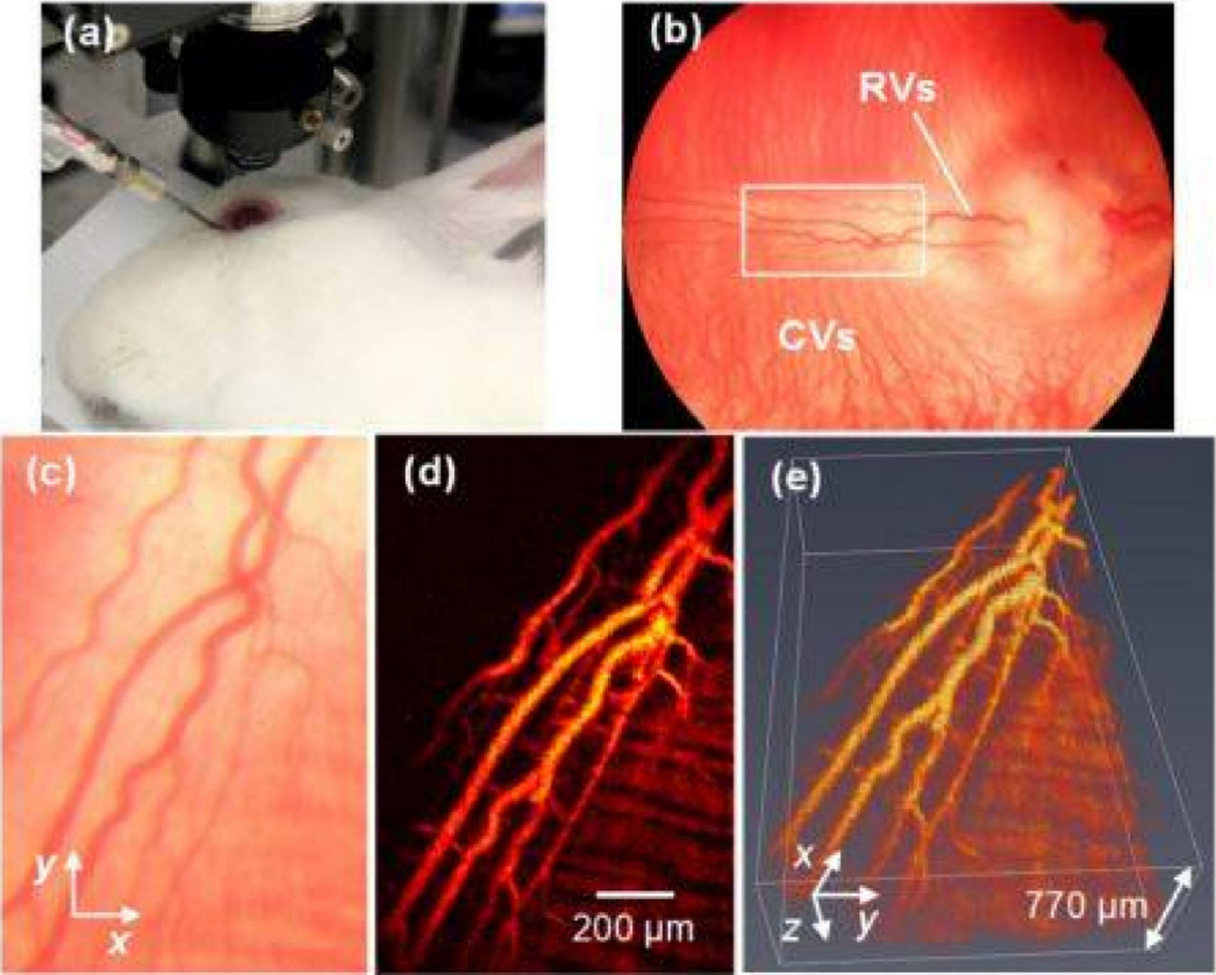
PAM imaging of retinal blood vessels in rabbits. (**a**) Experimental photograph showing the rabbit, the ophthalmic lens, and the ultrasound transducer; (**b**) Fundus photograph showing rabbit retinal vessels (RVs) originating from the optic nerve are confined in the medullary ray regions; (**c**) Close-up of the RVs in the white rectangle box in (**b**); (**d**) Maximum intensity projection (MIP) of PAM signals of RVs and choroidal vessels (CVs); (**e**) 3D volumetric rendering of the PAM image. Reproduced with permission from [60].

Multimodal imaging can be very beneficial for investigating ocular pathology and detecting disease. When PAM is integrated with other imaging modalities, such as OCT, fundus photography, ultrasound imaging, fluorescence imaging, confocal scanning laser ophthalmoscopy (cSLO), and multi-photon microscopy, more structural and functional information can be acquired [66–69]. The most useful integrated imaging system utilizes both multi-wavelength PAM and OCT. Tian et al. [68] reported a novel integrated PAM and SD-OCT to image the choroid, retina, and microvasculature in living rabbits (Figure 2). In this multimodal platform, OCT can provide structural information about the retina, and PAM can reveal functional and molecular details of biological tissue particularly when combined with nanoparticle contrast agents. Another multimodal imaging example is integrating PAM, SD-OCT, and autofluorescence-scanning laser ophthalmoscopy (AF-SLO) to visualize retinal vasculature and provide the concentration of melanin in the retinal pigment epithelium [70]. At present, PAM remains at an early stage in the development process. No clinically approved system exists for eyes, and all the photoacoustic imaging work in eyes has been performed in animals or in vitro. However, its development may greatly extend the scope of retinal imaging in future.

## 5. Adaptive Optics (AO) and Scanning Laser Ophthalmoscopy (SLO)

AO is an emerging discipline that seeks to improve the performance of an optical system by reducing the effects of wavefront distortions [71]. AO imaging systems use active optical elements to compensate for aberrations in the optical path between the object and the camera. AO systems have three principal components: a wavefront sensor, a corrective element, and a control system [72]. The power of AO is to provide cellular level resolution imaging of retinal cells by correcting for ocular aberrations. In recent years AO has been successfully integrated with some primary ophthalmic imaging devices, including AO-SLO, fluorescence AO-SLO, AO-OCT, and AO-two photon imaging. Each integration offers unique benefits [71].

**Figure 3.**
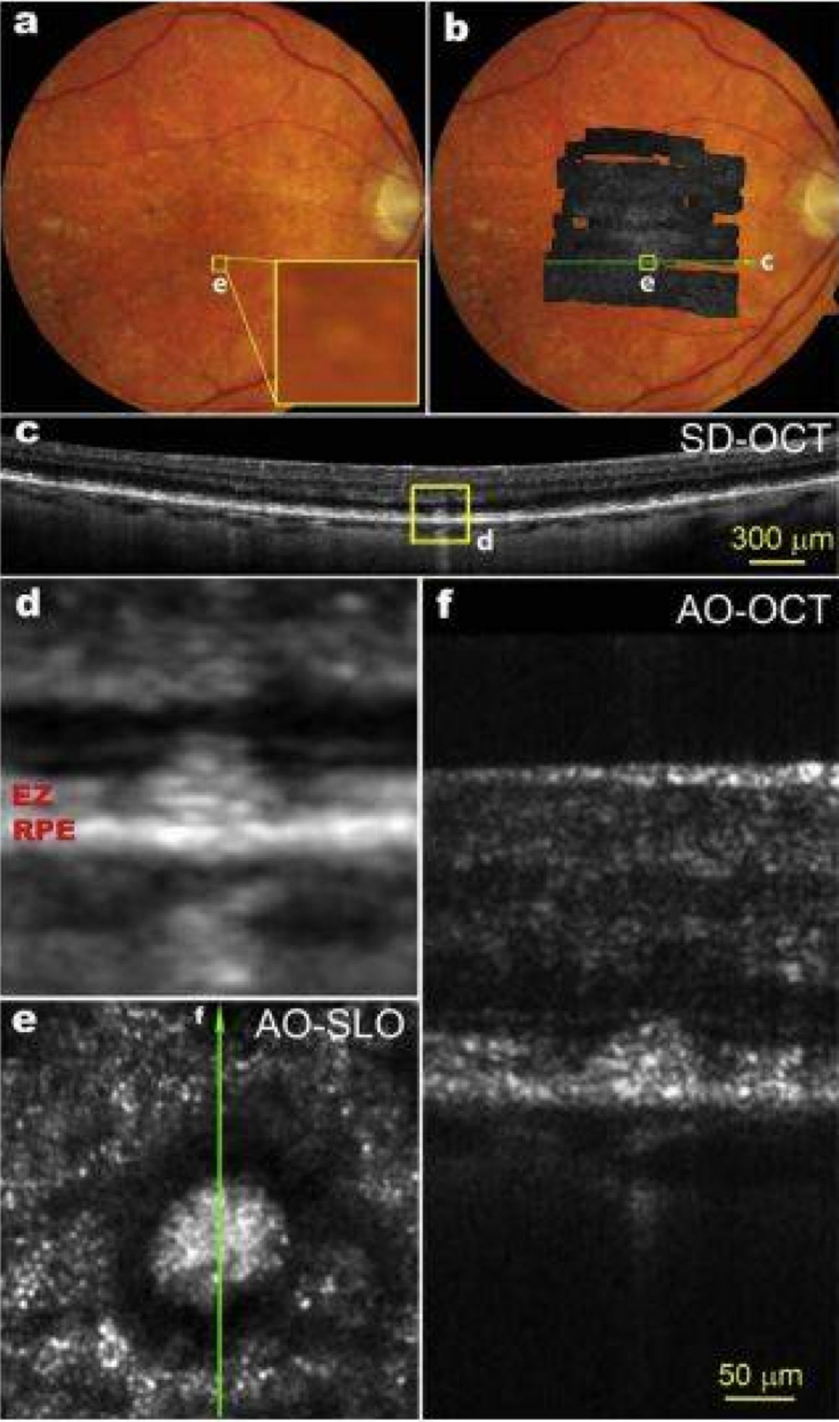
(**a**) Color fundus photograph of an 83-year-old patient with intermediate non-neovascular AMD; The yellow box (**e**) is 300 μm on each side; (**b**) AOSLO montage overlaid on the fundus photo; (**c**) A SD-OCT B-Scan taken along the green line in panel b shows that this subretinal drusenoid deposit (SDD) has broken the photoreceptor EZ band; (**d**) Magnification of boxed area in panel **c**; (**e**) The AOSLO image of the boxed retina in panels **a** and **b**. The bright spots outside the hyporeflective annuls are photoreceptors; (**f**) AO-OCT of the SDD, as indicted by the green line in panel **e**. Images (**d**–**f**) share the same scale bar. SD-OCT images are in logarithmic grey scale. AO-OCT is in linear grey scale [93].

Scanning laser ophthalmoscopy (SLO) was first described in 1981 [73]. Scanning laser ophthalmoscopy (SLO) uses a single, monochromatic laser with low power and a confocal raster scanning technique to collect an image of the retina and optic nerve head [58,73]. SLO images demonstrate higher contrast than standard fundus camera photos as they can reduce the effect of light scatter. SLO has been significantly improved through integration to AO, called AO-SLO [74]. AO-SLO systems have been reported to have the capability of observing individual cone and rod photoreceptors [75], blood vessels [76], capillaries [77,78], and RPE [79,80] Many of these same structures have also been visualized with AO-OCT imaging systems. 3D visualization of the retinal nerve fiber layer, microstructure in the ganglion cell layer [81] and Henle’s fiber layer, retinal microvasculature such as choriocapillaris and the capillaries that form the rim of the foveal avascular zone (FAZ), the 3D photoreceptor mosaic, the RPE, and the tiny pores of the lamina cribrosa of the optical nerve have been demonstrated using AO-OCT [72]. Both AO-SLO and AO-OCT are promising techniques (Figure 3). They are playing an important role in observing microstructures in living human retina with growing popularity. AO-OCT in particular offers some technical advantages over AO-SLO, including improved axial resolution and increased sensitivity to weak reflections [82].

Currently, imaging methods using AO are being applied to study several diseases including macular telangiectasia, cone-rod dystrophy, retinitis pigmentosa, and AMD. The ability of an AO ophthalmoscope to resolve cells and return to the same cells in the future offers the ability to track disease progression and monitor novel treatment strategies on an unprecedented cellular scale [83].

**Figure 4.**
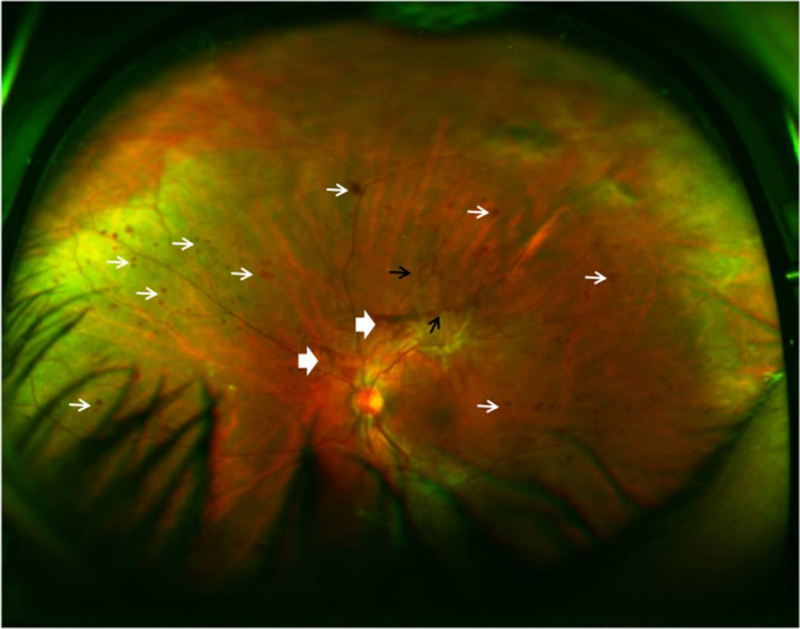
Ultra-wide field fundus SLO image (Optos, Dunfermline, UK) of a patient’s left eye with high risk proliferative diabetic retinopathy. White narrow arrow: severe intraretinal hemorrhages in 4 quadrants, and microaneurysms; white broad arrow: preretinal and vitreous hemorrhage; black arrow: retinal neovascularization.

Traditional fundus cameras take images of the retina with a 20- to 50-degree field of view. However, with conventional fundus images, a significant portion of the fundus remains unphotographed. Image montages were thus created to put together several photos and create a wider image of the retina. To extend the field of view, newer devices have sought to image more of the retinal surface, termed ultra-wide field imaging, and can provide up to a 200° view of the retina (Figure 4). This field of view is equivalent to 82% of the retinal surface compared to 15% offered by a single 45° image [84,85].

Current studies with ultra-wide field imaging modality have suggested important clinical applications for various retinal diseases, including DR, RVO, ophthalmic oncology, pediatric vitreoretinal disorders, and hereditary retinal degenerations. There is a growing consensus that ultra-wide field imaging improves detection of peripheral lesions in diabetic retinopathy, leads to more accurate classification of the disease, and gives important prognostic information on the diabetic retinopathy progression over time [85,86]. There is improvement in DR classification by using ultra-wide field imaging (Figure 4). Kumar et al. [87] described the use of ultra-wide field imaging in the diagnosis and management of adult onset Coats’ disease. They found that the ultra-wide field pseudo-color photographs and fluorescein angiograms were able to provide clinically useful information over and above that provided by conventional imaging. Ultra-wide field imaging has also been employed in other retinal imaging instruments, such as fluorescein angiography [88,89], OCT [90], SLO [91], and FAF [92].

## 6. Fundus Autofluorescence (FAF)

Fundus autofluorescence (FAF) imaging is a noninvasive imaging modality for in vivo mapping of naturally or pathologically occurring fluorophores of the ocular fundus. The primary sources are lipofuscin (LF) granules that have accumulated in retinal pigment epithelium (RPE) cells [94]. Excessive accumulation of lipofuscin granules in the RPE cells represents a common downstream pathogenic pathway in numerous retinal diseases [95]. The relevance of alterations in FAF images can further be addressed by assessing corresponding retinal sensitivity and response to stimuli. Severe damage to the RPE corresponds to areas of decreased autofluorescence.

The clinical applications of FAF continue to expand. It is an essential tool for evaluating AMD [96], geographic atrophy (GA), macular dystrophies, retinitis pigmentosa [97], white dot syndromes [98], and numerous other retinal disorders [99]. FAF imaging is particularly helpful for the differential diagnosis, detection, and extent delineation of involved retinal areas, genotype-phenotype correlation, and monitoring of changes overtime. FAF provides information on the metabolic state and overall health of the RPE and photoreceptors.

Quantitative AF (qAF) has potential applications in the diagnosis and monitoring of retinal conditions and may also have utility in prognosis and risk stratification. However, there is no universally accepted standard approach for qAF. This lack of standardized quantitative assessment can limit the utility of FAF as a method for diagnosis or monitoring and poses an important clinical need for future development.

## 7. Molecular Imaging

Conventional ophthalmic imaging platforms, such as fundus photography, scanning laser ophthalmoscopy, FA, ICGA, OCT, OCTA, and adaptive optics, are capable of imaging retinal anatomy and morphology with improved resolution. However, an important clinical challenge remains to visualize the early cellular and biochemical processes, which occur before advanced anatomic retinal changes. The aim of molecular imaging techniques is the visualization of molecular processes and functional changes in living animals and human patients before morphological changes occur at the cellular and tissue level [100]. Molecular imaging requires high image resolution, sensitive instrument detection, specific imaging agents, and endogenous molecular probes or exogenous contrast agents that link the imaging signal with a molecular probe or event [101,102].

Molecular imaging modalities include optical imaging techniques such as fluorescence and bioluminescence imaging, reflectance-based approach (e.g., SLO, retinal multispectral imaging, OCT), PAM, magnetic resonance imaging (MRI), radionuclide techniques such as positron emission tomography (PET) and single photon emission computed tomography (SPECT), ultrasonography, and computed tomography (CT) [100]. Several molecular imaging instruments are available, and all have their advantages and limitations. Multimodality molecular imaging combines instruments to utilize the advantages of modalities and to make up for the disadvantages of different modalities. So far, in vivo imaging of the ophthalmic molecular imaging has been largely applied in pre-clinical research using experimental animals by several research groups.

**Figure 5.**
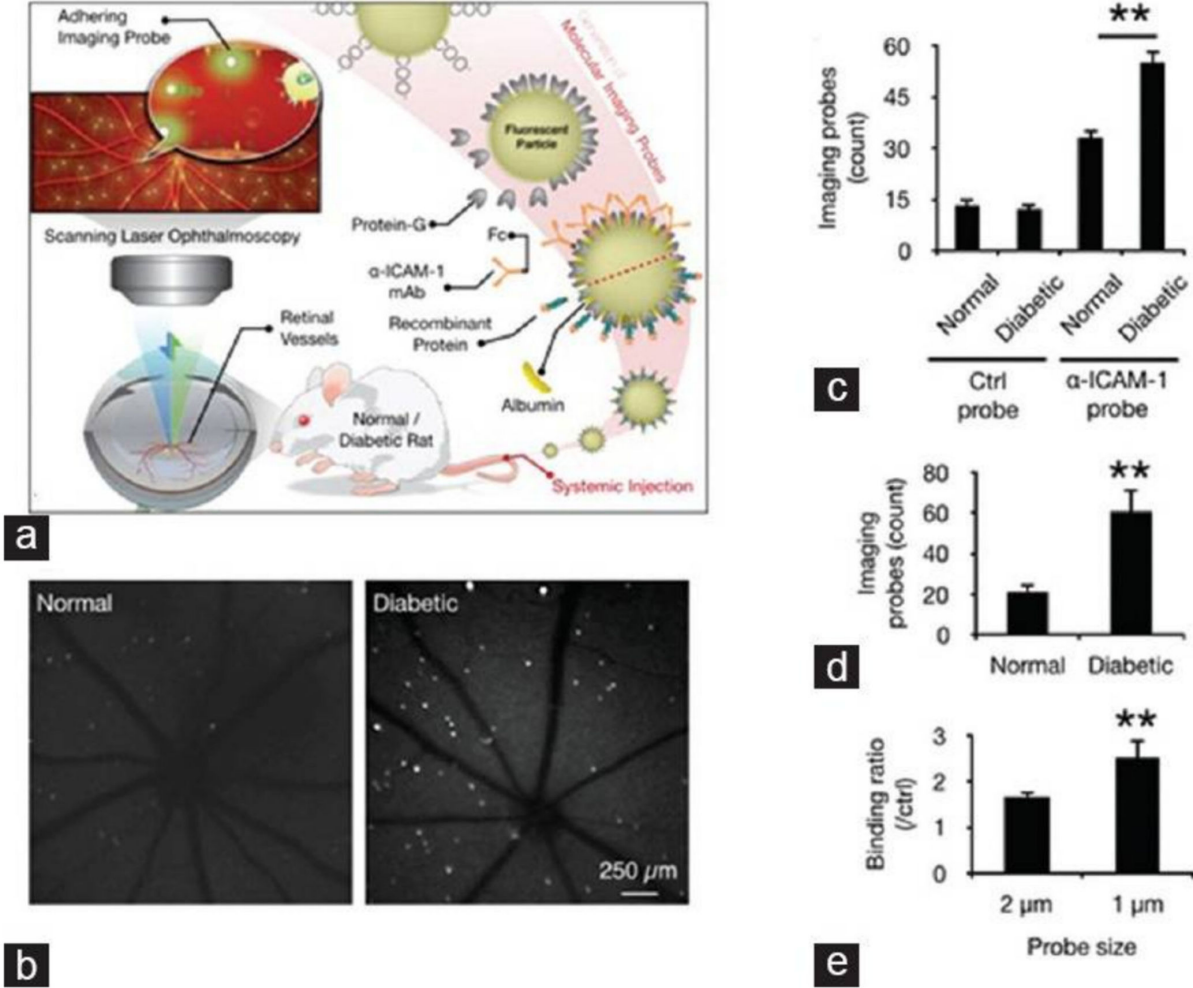
In vivo detection of endothelial injury using molecular imaging. (**a**) Schematic of in vivo molecular imaging approach; (**b**) Representative SLO-micrographs from the retinas of normal and diabetic animals. White dots represent firmly adhering probes; (**c**) In vivo probe adhesion in normal and three-week diabetic animals (*n* = 5, ** *p* < 0.01); (**d**) Molecular imaging of retinal endothelial ICAM-1 in 6 diabetic animals (*n* = 6, ** *p* < 0.01); (**e**) Comparison between the binding of two differently sized -ICAM-1 imaging probes (1 and 2 μm) in diabetic retinas (*n* = 5).

Molecular imaging depends on the development of specific and sensitive imaging agents, which is a pivotal step in the development of molecular imaging. Targeting moieties include small molecules, peptides, antibodies, and aptamers. These targeting moieties are applied to target imaging agents to recognize specific pathologic molecules [103]. Molecular probes are then detected. Molecular imaging is a potentially powerful tool for monitoring early events in retinal disease, such as apoptosis, cell injury, inflammation, and hypoxia. Frimmel et al. [104], in order to quantify the expression of retinal endothelial surface molecules in vivo, used α-ICAM-1 imaging probes to bind to ICAM-1. The results indicated that molecular imaging of retinal endothelial ICAM-1 could provide an early warning signal before clinical symptoms develop and can be used to detect subtle changes in the diabetic retina prior to the occurrence of irreversible pathology (Figure 5). Uddin et al. [105] suggest that, HYPOX-4, a new optical imaging probe, is capable of imaging retinal hypoxia in vivo in experimental RVO, before the onset of physiological changes leading to retinal cell damage and neovascularization. Tsuda et al. [106] injected molecular probe SYTOX orange (SO) into the vitreous of optic nerve crush (ONC) mice. With this, retinal ganglion cell (RGC) death was visualized with a confocal scanning laser ophthalmoscope (cSLO) in vivo. The study concluded that real-time imaging with SO was able to quickly quantify ONC-induced RGC death. This method may be an effective way to understand the pathogenesis of diseases involving RGC death, particularly glaucoma and to monitor disease progression. Recently, a new cutting–edge technology called “Detection of Apoptosing Retinal Cells” (DARC) has been reported to detect RGC apoptosis in vivo [107,108]. Annexin V is labeled with a fluorescent marker, such as FITC, bound to phosphotidylserine has been utilized as a sensitive probe for the identification of apoptosing cells [109,110]. The apoptosing cells can then be visualized using conventional ophthalmic imaging devices such as cSLO. It can detect RGC damage at a very early stage, at the moment apoptosis starts, even before visual field defects develop.

Recently, the application of nanotechnology has stimulated the development of imaging agents. Nanoparticles modified with unique and tunable optical properties, along with their small size and capacity for cellular targeting, make them as promising class of molecular imaging agents. It has been reported that gold nanoparticles have the potential for use as contrast agents with OCT. With multimodality imaging techniques clearly on the rise, the development has led to explosive growth in multimodal imaging agents [111]. Nanoparticles are attractive candidates for multimodal imaging probes [112]. Although there are few reports on the application of nanoparticles to ophthalmic multimodality imaging systems, molecular imaging is rapidly expanding and showing significant promise. With the ongoing advancement in theranostics, molecular imaging should not only be viewed solely as an imaging tool, but instead as a possible platform for the clinical application of nanotherapeutics.

While preclinical molecular imaging is useful in the development of nanomaterials or other agents, clinical molecular imaging is critical for the appropriate application of nanomaterials. Nevertheless, many critical challenges, including toxicity, biocompatibility, targeting efficacy, and long-term stability of nanomaterials, should be addressed for their clinical translation. It is also important to choose the most suitable nanomaterials and imaging modalities to obtain desired information. The sustainable development of nanotechnology in molecular imaging is expected to drive the next generation of diagnosis and therapy of diseases in the future.

## 8. Current Clinical Challenges and Future Directions in Retinal Optical Imaging

While optical imaging can provide unprecedented image resolution and speed, clinical challenges persist in the field. One clinical challenge is the lack of adequate biomarkers. Current imaging-based clinical biomarkers do not provide an adequate correlation between anatomy and function. For example, trials of anti-vascular endothelial growth factor therapy have demonstrated that visual acuity does not correlate with OCT thickness measurements. Also, OCTA reveals vasculature but has difficulty visualizing microaneurysms and leakage, the two primary reasons to acquire fluorescein angiograms. While we have seen great improvements in vascular imaging (e.g., OCTA), there remains limited ability to evaluate ischemia and hypoxia in tissue micro-environments. Several diseases can impede the ability of tissue to extract oxygen and nutrients even when present within vasculature, and thus moving from vascular analysis to extravascular tissue-level analysis becomes critical. While SLO and ultra-wide field imaging have improved our visualization of the periphery, there remain challenges in visualization of the far periphery and thus systems that provide an even wider field of view from ora serrata to the ora serrata are needed. Improved visualization and understanding is also needed of the choroid, which is being noted to play an increasingly important role in retinal diseases. There is also need for imaging the retina in patients with media opacities (e.g., vitreous hemorrhage, dense white cataracts, severe central keratitis, and total 8-ball hyphemas) where currently we can only perform low resolution 10 MHz B scan ultrasonography clinically.

Another challenge is the rapid interpretation and analysis of the large amount of data generated from these imaging modalities. With declining reimbursements, particularly for imaging, the amount of time retina specialists spend per patient has been reduced, with many physicians spending 5 to 10 min per patient to obtain a clinical history, examine, evaluate and interpret imaging, answer patient questions, educate patients, document, and treat patients. Thus, there is a need for computer-assisted interpretation and software to facilitate the rapid evaluation of the extensive data generated from these images. In addition, there is a clinical shortage of skilled and trained ophthalmic photographers, particularly in developing countries, and thus it is critical that new devices be developed that can be performed by minimally trained allied health care professionals without extensive training. While most imaging requires large table-top systems, there is further need for mobile imaging platforms, such as hand-held OCT and intra-operative OCT.

Medicine, retina, and therapies are increasingly driven by molecular and genetic changes, and thus there is a major clinical need for non-invasively determining molecular and genetic changes taking place in tissue. While one can acquire blood, aqueous humor, and even vitreous readily, getting tissue access of retina through biopsy is challenging with a significant risk of complications. Thus, there is a need for non-invasively measures of retina molecular markers to evaluate ischemia, inflammation, cell injury, and cell death which could necessitate multi-modal imaging platforms. There is also significant need for theranostics, where agents can both serve as diagnostic and therapeutic modalities.

## 9. Discussion/Conclusions

Optical imaging has played an indispensable role in giving us our current understanding of retinal and choroidal disease. We currently stand on the cusp of a revolution in retinal optical imaging with numerous recent advances, including OCT, OCTA, adaptive optics, SLO, fundus autofluorescence (FAF), photoacoustic microscopy (PAM), and molecular imaging. These imaging modalities have begun to transform our understanding of the molecular pathogenesis of retinal disease and are playing an increasing role in the early diagnosis and management of patients. Continuous innovations in imaging technology and progress in the understanding of retinal pathophysiology will make optical imaging continue to play a critical role in retinal diseases for many years to come.
